# The Environmental Impacts of Radio Frequency and Power Line Communication for Advanced Metering Infrastructures in Smart Grids

**DOI:** 10.3390/s23249621

**Published:** 2023-12-05

**Authors:** Ons BenRhouma, Chiheb Rebai, Manel Ben-Romdhane, Dario Di Cara, Giovanni Artale, Nicola Panzavecchia

**Affiliations:** 1 COSIM Research Laboratory, SUP’COM, University of Carthage, Ariana 2083, Tunisia; chiheb.rebai@supcom.tn (C.R.); manel.benromdhane@supcom.tn (M.B.-R.); 2 Institute of Marine Engineering (INM), National Research Council (CNR), 90146 Palermo, Italy; nicola.panzavecchia@cnr.it; 3 Department of Engineering, Università degli Studi di Palermo, 90128 Palermo, Italy; giovanni.artale@you.unipa.it

**Keywords:** power line communication, RF mesh, urban area, industrial area, rural area, electric grids, sensing and measurement, BER

## Abstract

In the neighborhood area network (NAN), the advanced metering infrastructure (AMI) enables a bidirectional connection between the smart meter (SM) and the data concentrator (DC). Sensors, such as smart meter nodes or environmental sensor nodes, play a crucial role in measuring and transmitting data to central units for advanced monitoring, management, and analysis of energy consumption. Wired and wireless communication technologies are used to implement the AMI-NAN. This paper delves into a novel approach for optimizing the choice of communication medium, air for radio frequency (RF) or power lines for power line communication (PLC), between the SM and DC in the context of the AMI-NAN. The authors methodically select the specific technologies, RF and NB-PLC (narrowband power line communication), and meticulously characterize their attributes. Then, a comparative analysis spanning rural, urban, and industrial settings is conducted to evaluate the proposed method. The overall reliability performance of the AMI-NAN system requires a packet error rate (PER) lower than 10%. To this end, an efficient approach is introduced to assess and enhance the reliability of NB-PLC and RF for AMI-NAN applications. Simulation results demonstrate that wireless communication is the optimal choice for the rural scenario, especially for a signal-to-noise ratio (SNR) lower than 25 dB. However, in urban environments characterized by higher SNR values and moderately dense networks, NB-PLC gains prominence. In denser networks, it outperforms wireless communication, exhibiting a remarkable 10 dB gain for a bit error rate (BER) of 10^−3^. Moreover, in industrial zones characterized by intricate network topologies and non-linear loads, the power line channel emerges as the optimal choice for data transmission.

## 1. Introduction

A neutral grid represents a hypothetical concept and a theoretical extension of smart grid technologies, incorporating machine learning capabilities and advanced artificial intelligence [1]. Its main objectives are sustainable energy integration, advanced energy management capabilities for consumers, energy data security, and real-time data prediction. To achieve these objectives, it is needed to collect data related to many operations such as production, storage, consumption, distribution, and transmission of electricity relying on its communication infrastructure. Accordingly, communication infrastructure of the smart grid must cover a very large geographical area, which may range from distant production sites to overcrowded residential areas, namely buildings and houses.

For smart metering applications, such as smart home/building, energy management, grid monitoring, and integration of renewable energy sources (RES), several communication technologies can be employed, such as NB-PLC [2], BB-PLC [3], Zigbee [4], LTE, UMTS [5], GSM, WiFi, etc. [6,7]. Table 1 provides an overview of the most used and suitable wired and wireless technologies for low-voltage (LV) smart metering applications.

Among the available technologies, NB-PLC for wired and RF mesh for wireless technologies demonstrate significant promise for efficiently allocating energy resources in smart metering applications. The choice between RF and NB-PLC depends on factors such as coverage requirements, environmental conditions, interference levels, and specific application needs within the smart grid domain. However, despite their cost-effectiveness for advanced metering infrastructure (AMI) applications, NB-PLC and wireless channels present challenges in terms of reliability and overall communication system performance. This paper focuses on investigating the communication between the data concentrator (DC) and the smart meter (SM) in three selected areas: rural, urban, and industrial. To address this objective, the authors engage in standardization efforts, considering two solutions: (1) incorporating functioning standards into the existing electricity grid with necessary modifications, and (2) developing new communication protocols tailored to the communication requirements of the smart grid.

For wireless links, IEEE 802.15.4g [8] and IEEE 802.11 ah [9] can be used. In this paper, the authors choose the IEEE 802.15.4g standard. In fact, the wireless communication standard Wi-SUN (wireless smart utility network) enables seamless connectivity between smart grid devices and can be implemented with extremely low levels of power consumption compared to other wireless communications. Its physical layer is based on the IEEE 802.15.4g technology operation in the 863 MHz–870 MHz ISM band. Moreover, Wi-SUN allows utilities to monitor and control the electrical grid more efficiently, leading to improved energy management and reliability. It supports mesh networking, allowing devices to communicate with each other via multiple paths. However, in some cases, long-range and licensed standards can be used to obtain a higher data rate and lower latency for some types of collection of critical data between smart meters and data concentrator. Sometimes, a mix between Wi-SUN and LTE-M can be offered as in smart city street lighting, where integrating the two standards can provide a robust communication network for managing and controlling streetlights [10]. On the one hand, the deployment of Wi-SUN creates a local mesh network for communication between streetlights. On the other hand, the use of LTE-M for backhaul communication allows to remotely control streetlights in less densely populated areas where Wi-SUN coverage may be limited. In this paper, the authors focus their study on Wi-SUN (based on IEEE 802.15.4g) as it is unlicensed, is a low-power technology, and has long-range capabilities.

For wired links, several works have been focused on PLC standardizations. For narrowband PLC transmissions, the well-known standards are G3-PLC, PRIME [11], G.hnem [12], and IEEE 1901.2 [13]. In [14], a physical layer standard performance of the three standards, G3, PRIME and IEEE 1901.2, were compared. In [15], the authors proposed a new architecture and new devices for distributed generation (DG) and energy storage system (ESS) management using PLC technology. The architecture was developed in the framework of the SInERT project, financed by the Italy–Tunisia cross-border cooperation program 2014–2020. The project aims at proposing innovative solutions to integrate renewable energy on the Tunisian electrical grid. In particular, this study included tests on PRIME [16] and G3-PLC [17], demonstrating the feasibility of the proposed solution for remote monitoring and control of DG and ESS. In this paper, the authors further investigate the PLC solution, particularly the IEEE 1901.2, which is a revision of G3-PLC operating in the frequency band from 3 kHz to 500 kHz [18,19,20]. In fact, G3-PLC offers several advantages when compared with other PLC standards as it provides long-range communications and cost-efficient LV power grids. Moreover, it is designed to operate reliably in impulsive and noisy channel conditions and ensures robust communication. IEEE 1901.2 is a standard for narrowband orthogonal frequency division multiplexing (NB-OFDM) power line communication (PLC) technology. The use of OFDM, which is known for its ability to handle multipath propagation and mitigate the effects of noise and interferences, makes a robust communication in challenging environments, such as industrial ones.

Many research papers are interested in evaluating communication performance in AMI infrastructures. In [21], authors analyzed communication performances and measured the signal strength and the noise power at different measured and computed the packet error rate (PER). In [22,23], authors analyzed security vulnerabilities, attacks, and countermeasures for AMI applications. In [24,25], bit error rate performances (BER) were quantified for a PLC channel using OFDM modulation and a hamming code in the presence of additive white Gaussian noise (AWGN) and impulsive noise. However, in this paper, the authors designed an AMI-NAN solution that selected an appropriate link, RF, or PLC to guarantee an uninterrupted communication between smart meters and data concentrators. This greatly depended on the BER, which impacts the PER and depends on the distance between the SM and DC. Moreover, data rates vary depending on factors such as the specific implementation, the quality and conditions of power lines, and the presence of interferences. Indeed, experimental results and channel models are applied to the Wi-SUN channel simulation for different values of data rates [26]. Therefore, it is necessary to understand the distinct channel characteristics of communication links to enhance the performance of any installed communication scheme. Hence, studying these characteristics is essential in order to develop optimal communication solutions and ensure system reliability. This involves selecting the most suitable channel, whether it is PLC or RF, across various topologies, such as rural, urban, and industrial settings, as well as different noise profiles.

**Figure 1:**
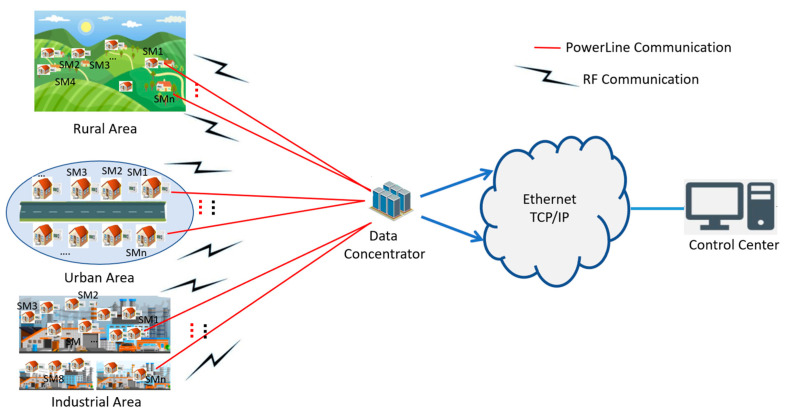
General system overview.

In this regard, the main objective of this paper is to establish a novel approach for ensuring uninterrupted communication between the SM and DC in the advanced metering infrastructure (AMI) system, with a focus on achieving optimal performances in terms of the BER across three distinct environments, as illustrated in Figure 1. The proposed method outlines a dependable communication system that relies on a NB-PLC medium when the wireless signal is compromised or weakened due to obstacles or disruptions, and on a wireless medium when the PLC signal is negatively impacted by excessive noise or an impedance mismatch.

In Section 2, the authors outline the key characteristics of RF and NB-PLC systems. Section 3 provides an overview of various environments: rural, urban, and industrial. In Section 4, the impacts of these environments are analyzed through simulations using both NB-PLC and RF systems. The parameters for each system are set based on IEEE 1901.2 for NB-PLC and IEEE 802.15.4g for RF. The results demonstrate the appropriateness of each link in relation to a specific scenario, considering factors such as multipath effects and the type of noise as indicators of channel performance.

## 2. NB-PLC and RF Characterization

RF and NB-PLC technologies are both employed in AMI-NAN systems to facilitate the communication between different devices and components. In this section, the authors provide a characterization of each technology, specifically in terms of attenuation and noise.

### 2.1. NB-PLC Characterization

PLC suffers from harsh conditions such as unexpected variations in channel attenuation, multipath effects, and uncommon noise characteristics [27]. In this section, the authors overview NB-PLC attenuation and its noise characteristics.

#### 2.1.1. NB-PLC Attenuation

**Figure 2:**
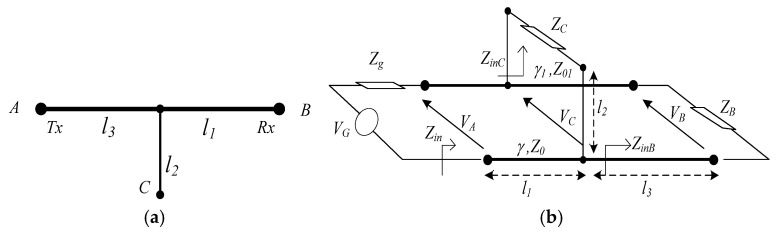
(**a**) Single branch; (**b**) elementary branch parameterization.

The NB-PLC signals transmitted on power lines are affected by high attenuation due to the propagation of line losses. These depend on the topology of a network, environmental conditions, and behavior of connected appliances, exhibiting variable spectro-temporal characteristics [28]. As a function of loads and of a network’s layout, PLC may induce high attenuation and deep fading effects. The generation of multipath signals is a result of impedance mismatches, which are caused by the presence of many loads connected to a network’s termination points and multiple cable branches. Therefore, the reflected waves yield to multiple delayed echoes in the channel impulse response. In [29], authors develop a delay detection technique for the PLC multipath channel and examine the impact of the electric line length, number of branches, and impedances on the channel path delays. In [30], the authors investigate NB-PLC channel modeling and present a bottom-up approach, namely the voltage ratio approach (VRA), which is based on the topology of a network. A comparison of different NB-PLC models is presented in [31], in which it is demonstrated that the PLC transfer function depends on the network topology and the type of connected loads. Thus, in this paper, the authors opt to apply the VRA model as it accurately computes the transfer function. A single branch is defined in Figure 2a as the combination of three points: A is the transmitting point (Tx), B is the receiving point (Rx), and C is the connected load which comprises another branch. The equivalent representation of a branch in terms of transmission line (TL) parameters is depicted in Figure 2b. In the representation in Figure 2b, *l*_1_ denotes the cable length from the Rx end of the path line to the tap point C, *l*_2_ denotes the length of the cable connected from the tap point C to the backbone, and *l*_3_ represents the cable length from the tap point C to the Tx end of the path line A.

The input impedances, *Z_inB_*, *Z_inC_*, and *Z_in_*, are determined using Equations (1) and (2). Without the loss of generality, the authors assume that the direct backbone path (from A to B) consists of the same transmission line with a defined propagation *γ* and characteristic impedance *Z*_0_. The stub *Z_C_* is represented by a different line with a defined propagation constant *γ*_1_ and characteristic impedance *Z*_01_.

(1) $$Z_{inB}=Z_{0}\frac{Z_{B}+Z_{0}\tanh(\gamma l_{3})}{Z_{0}+Z_{B}\tanh(\gamma l_{3})}\;\mathrm{and}\;Z_{inC}=Z_{01}\frac{Z_{C}+Z_{01}\tanh(\gamma_{1}l_{2})}{Z_{01}+Z_{C}\tanh(\gamma_{1}l_{2})}$$

(2) $$Z_{in}=Z_{0}\frac{\left(Z_{inB}//Z_{inC})+Z_{0}\tanh(\gamma l_{1}\right)}{Z_{0}+(Z_{inB}//Z_{inC})\tanh(\gamma l_{1})}$$

In order to calculate the transfer function of a basic branch, the reflection coefficients of the end path (Γ_1_) and from the tap point (Γ_2_) are introduced. By applying the VRA method between access point A and end point B, the transfer function *H_e_*(*f*) is given in Equation (3) as follows:

(3) $$H_{e}\left(f\right)=\frac{Z_{in}}{Z_{in}+Z_{g}}\times \frac{\left(1+\Gamma_{1}\right)e^{-\gamma l_{3}}}{1+\Gamma_{1}e^{-2\gamma l_{3}}}\times \frac{\left(1+\Gamma_{2}\right)e^{-\gamma l_{1}}}{1+\Gamma_{2}e^{-2\gamma l_{1}}}$$

For multibranch network and using (1), the transfer function is written in Equation (4) as follows:

(4) $$H_{e,network}\left(f\right)=\overset{N}{\underset{k=1}{\prod }}H_{e}^{\left(k\right)}\left(f\right)$$

where *N* represents the number of branches in the network and $H_{e}^{\left(k\right)}\left(f\right)$ is the transfer function of the elementary branch.

**Figure 3:**
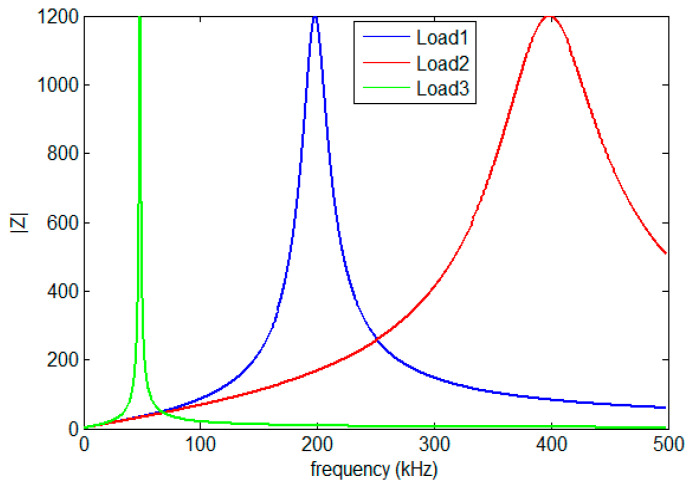
Behavior of loads.

In this paper, the authors suppose that the network between the transmitter and the receiver is a cascade of three single branches where a cable’s length depends on the area type: rural, urban, or industrial. Loads are frequency-selective and modeled using a resonant RLC circuit. The input impedance *Z*(*f*) is given in Equation (5) as follows:

(5) $$Z\left(f\right)=\frac{R}{1+j.Q.\left(\frac{f}{f_{0}}-\frac{f_{0}}{f}\right)}\;\;$$

where *R* is the resistance, *Q* is the factor quality that determines selectivity, and $f_{0}$ is the resonance frequency. Examples of the behaviors of used loads are depicted in Figure 3.

**Figure 4:**
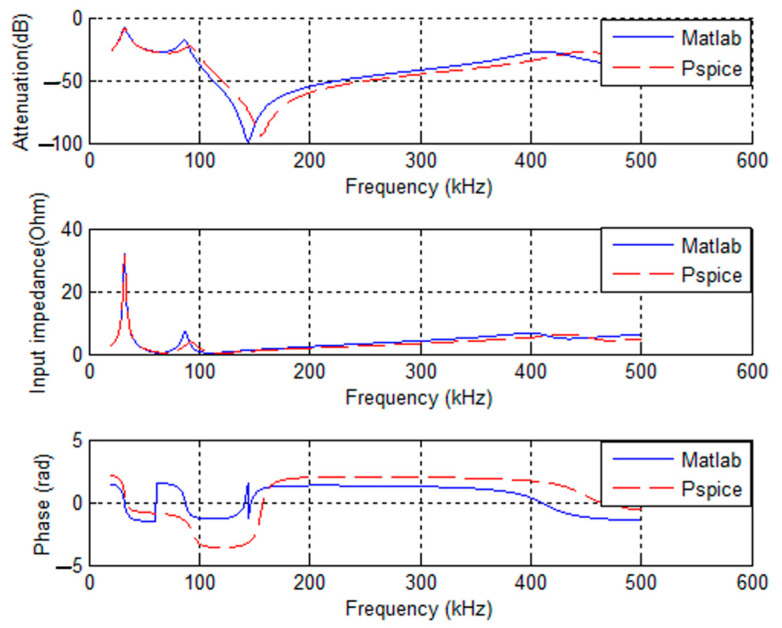
Example of NB-PLC behavior.

Based on the VRA method, an analytic model for industrial area is developed using Equation (3) and implemented in the R2014a Matlab tool. This model is then compared to the circuit model in Pspice, a CAD tool, for a three-core cable consisting of phase, neutral, and ground branches and when the cable type is assumed to be the same for the three branches. The simulation results obtained from both models are illustrated in Figure 4. The curves for attenuation, impedance, and phase exhibit non-smoothed traces. These irregularities arise due to the presence of discontinuities at various frequencies, resulting in the reflection of propagating waves and power loss.

**Figure 5:**
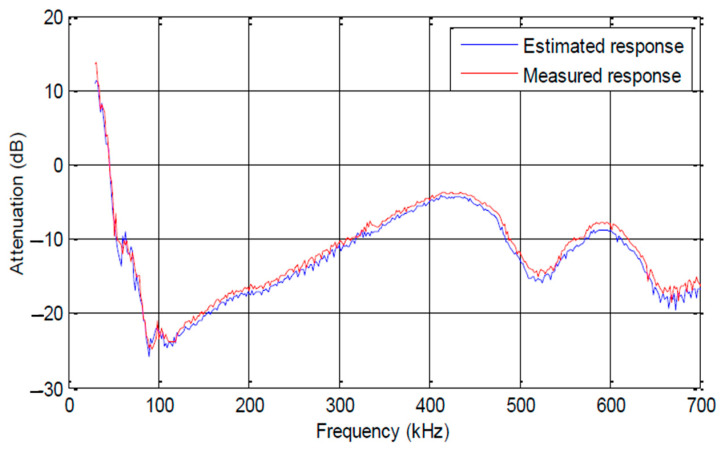
Measurement versus simulation of NB-PLC channel.

In [32], a coupling interface was implemented to build a measurement platform, facilitating a comparison of simulated results. This interface serves two major roles: The first is instrument protection. The second is the injection or extraction of the test signal to or from the mains. Then, a two-step verification methodology was proposed using the bottom-up approach. Measurements were performed in a real environment where many computers were plugged, as well as spectrum analyzers. All the measurement setups are connected to the same line phase. The test circuits were constructed using both 1.5 mm^2^ and 2.5 mm^2^ core cables, with each section spanning a length of 1 m. For NB-PLC noise, the authors opted to use the cyclostationary-based filter model already published in [33]. The impact of some events on the network is shown either by a translation of the channel response magnitude or by an attenuation at distinct frequencies. Figure 5 shows that simulated transfer function magnitude agrees with the measured one, with an error lower than 3 dB.

#### 2.1.2. NB-PLC Noise

**Figure 6:**
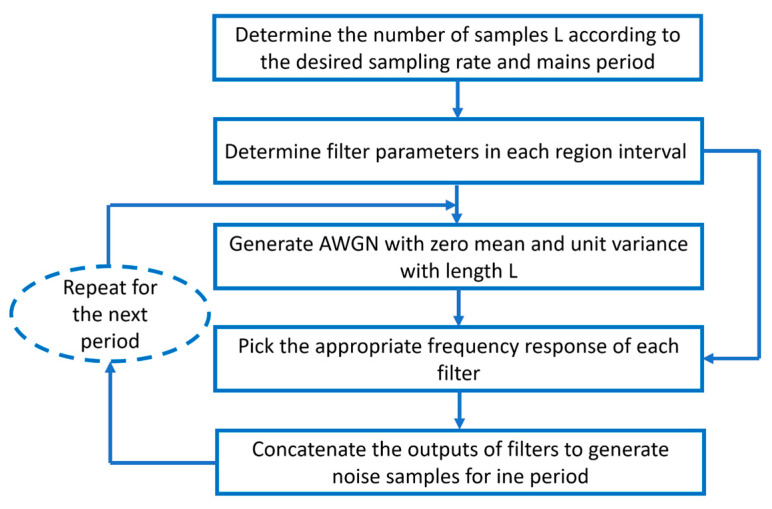
Flowchart of periodic impulsive noise generation.

According to [34,35,36], PLC noise may be classified into three types: (1) periodic impulsive noise characterized by its cyclostationarity in both the time and frequency domain; (2) background noise where its power spectral density (PSD) decreases exponentially; and (3) the asynchronous impulsive noise known as random impulses. Since the periodic impulsive noise is the dominant noise component of NB-PLC, authors select it to be the noise model for the power line channel. In this paper, following the IEEE 1901.2 standard, the linear periodical time-varying (LPTV) system models the periodic impulsive noise [37]. Given this model, the noise samples are written as in Equation (6) as follows:

(6) $$n_{k}=\overset{N}{\underset{i=1}{\sum }}1_{k\in R_{i}}\underset{\tau }{\sum }h_{\tau }^{\left(i\right)}\upsilon_{k-\tau }\;\;\;\;\;$$

where 1*_k_* is the indicator function, $h{\;}_{\tau }^{\left(i\right)}$ is the impulse response of the LTI filter in the interval *R_i_*, and *v_k_* obeys the Gaussian distribution. The generation of periodic impulsive noise process is summarized in Figure 6. First of all, the authors determine the number of L samples according to the desired sampling rate and mains period. Then, they determine the parameters of each filter corresponding to each region interval. Afterward, a generation of L-length AWGN with zero mean and unit variance is essential to pick the appropriate frequency response of each filter. Finally, the filters’ outputs are concatenated in order to generate noise samples for one period.

**Figure 7:**
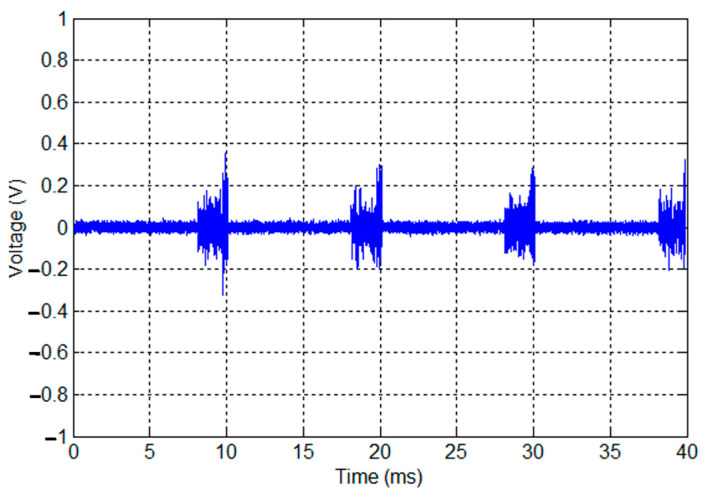
Cyclostationary noise wave.

In Figure 7, the authors present the periodic impulsive noise wave for four periods. This type of noise is essentially characterized by three parameters: number of regions (N_R_), region interval (R_j_), and the LTI filters. All three parameters are parametrized by their corresponding noise PSDs. The authors summarize the percentage of each region’s intervals in Table 2.

According to Table 2, the first region corresponds to the biggest number of OFDM symbols. This corresponds to the duration of the first filter (6.6 ms) used for noise shaping in the AC cycle being the highest compared to the two regions R_2_ (1.38 ms) and R_3_ (0.26 ms).

### 2.2. RF Characterization

In smart metering applications, the RF channel model describes the behavior of wireless communication channels between smart meters and data concentrators or other points within a smart grid system. This channel model provides a mathematical representation of RF signal propagation and interaction with the environment, besides the various impairments and effects during transmission. Understanding and modeling the attenuation and the noise of the RF channel is crucial for designing and optimizing communication systems to ensure reliable data transmission and reception in smart metering networks.

#### 2.2.1. RF Attenuation

In this paper, the authors are interested in outdoor environments (rural, urban, and industrial) where the wireless signal is subject to several fading phenomena, such as reflection, refraction, diffraction, etc. Channel modeling is one of the common challenges for the Wi-SUN mesh network [38]. This process is primarily employed to predict signal characteristics including channel behaviors such as propagation phenomena (absorption, reflection, scattering, and diffraction), interference sources (involving thermal noise and shared unlicensed frequency bands), and the dynamic nature of the environment (multipath fading channels). By solving Maxwell’s equations for all physical elements and environmental factors, the channel model is obtained, acquiring multipath fading channels. Consequently, a pragmatic approach involves focusing the channel model on the primary propagation effects, namely reflection, diffraction, and scattering, while disregarding less pertinent details to streamline complexity. These fading phenomena are categorized into two distinct groups: large-scale fading and small-scale fading. Large-scale fading characterizes the gradual alteration of signals, primarily attributed to the combined effects of path loss and shadowing. Oppositely, small-scale fading pertains to the swift fluctuations of signals on a smaller scale, driven by three fundamental factors: the signal’s delay spread, temporal variations within the channel, and the transmission bandwidth of the channel. For smart metering applications, some of the key components and considerations in an RF channel model include path loss and multipath propagation.

Path loss, expressed in dB, refers to the attenuation or reduction in the strength of a wireless signal as it propagates through space over a certain distance. It is a critical factor because it directly affects the reliability of wireless communication links between smart meters and data concentrators. Authors in [39] estimated the connectivity of a smart meter and a data concentrator using a more detailed approach. They employed a path loss model, which includes link-specific terrain profile information to compute diffraction losses. This allows for a better estimation of the average link-received power for the complete path between each SM and the DC around it. Smart meters are typically deployed across various environments, such as rural, urban, and industrial areas. Hence, path loss can vary significantly based on factors such as distance, operation frequencies, and obstacles. For outdoor environments, its typical values vary from 2.7 to 6.5 depending on the environment characteristics [40]. In [41,42], research studies have demonstrated that the path loss exponent is typically observed to be around 4 for urban areas. In industrial areas, the path loss exponent tends to be around 3, indicating a slightly lower rate of signal attenuation compared to urban areas. On the other hand, flat-lying rural areas generally exhibit even lower values of the path loss exponent. These variations in the path loss exponent reflect the different characteristics of each environment and the impact of obstacles, terrains, and other factors on signal propagation.

The multipath propagation occurs when signals take multiple paths between the SM and the DC, leading to constructive or destructive interferences. Interference emerges from the fusion of signals arriving from diverse directions—a direct path, reflections, diffractions, and scatterings—each accompanied by random amplitudes, phases, and delays. This causes fading and impacts signal strength and the SNR or BER. Therefore, the complex nature of wireless communication channel behavior requires well-constructed channel models for an accurate analysis and network optimization. The Rayleigh fading model is adopted, and it shows the effects of small-scale fading of a non-line-of-sight channel in wireless communications. A multipath wireless channel can be modelled using the method of the impulse response with the Equation (7) [43] as follows:

(7) $$h\left(t,\tau \right)=\overset{N\left(t\right)}{\underset{i=1}{\sum }}a_{r}\left(t\right)e^{j\theta_{r}\left(t\right)}\delta \left(\tau -\tau_{r}\left(t\right)\right)$$

where $a_{r}\left(t\right)$ is the amplitude, $\theta_{r}\left(t\right)$ is the phase, N(t) represents the number of paths which depends on *t*, and $\tau_{r}\left(t\right)$ is the delay.

#### 2.2.2. RF Noise

In addition to the AWGN, a major design challenge for wireless transmission on unlicensed bands is the presence of strong interference produced primarily by uncoordinated transmissions and neighboring impractical devices on the same frequency band.

Such interference is characterized by three statistical models of impulsive noise: (1) the stable alpha-symmetric variable (SαS), (2) the Middleton class-A (MCA), and (3) the Gaussian mixture (GM). The symmetric stable alpha-unsystematic variable is similar to the GM model, and the MCA probability density function (PDF) is a special case of the GM PDF. In the literature, to the best of the authors’ knowledge, there are no works that present noise modeling in the 863–870 MHz wireless frequency band. Assuming the analogy between the sub-1 GHz and the 2.4 GHz band, authors in [44] model noise as a two-component Gaussian mixture random process and obtain the best distribution that fits the major source of RF interference (RFI) in a wireless channel from the RFI toolbox [45].

Therefore, the probability density function of the GMM (Gaussian mixture model) is computed as a summation of complex Gaussian distributions with zero mean and unit variance $\sigma_{k}^{2}$ and is given in Equation (8) as follows:

(8) $$f\left(x\right)=\overset{K}{\underset{k=1}{\sum }}\pi_{k}N_{c}\left(x|0,\sigma_{k}{}^{2}\right)\;\;$$

where $\pi_{k}$ is the mixing probability and $\sigma_{k}$ is the variance of the *k*th Gaussian component.

Both RF and NB-PLC technologies have their advantages and drawbacks, and the choice between them depends on factors such as the specific requirements of the utility company, the geographical characteristics of the environment, and regulatory considerations. In the next section, the authors present the environment characterization.

## 3. Environment Characterization

Environment characterization is a critical step in AMI applications, as it ensures that a given communication technology is optimized under specific conditions of the environment, providing reliable and accurate data for smart metering and energy management. Communication between the SM and DC covers a very large geographical area, with its characteristics strongly affecting the transmitted signal for wired and wireless technologies. In fact, dense obstructions, such as buildings, floors, walls, etc., or new constructions or tree growth heavily attenuate wireless signals. However, the PLC signal depends only on the electric network topology and any modification or extension of such network may damage the existing communication. Therefore, to efficiently extend the environment limitation and transmit data to the destination through two different characteristics of communication technologies, wired and wireless, the authors classify areas based on two criteria: the density of loads and the density of obstructions. This section defines the three environments, namely rural, urban, and industrial.

### 3.1. Rural Environment

The rural environment is categorized into a low-density area. In fact, it refers to an outside area of urban or suburban regions that is primarily characterized by a natural or agricultural landscape. Both wired and wireless communication technologies are used in rural environments to provide access to communication and information resources. Each technology has its own advantages. The best choice depends on the specific needs and circumstances of the utilities.

PLC technology is a useful technology in rural areas where power lines already exist. It provides a reliable and cost-effective way to transmit data and communication signals over long distances. However, PLC is limited by the quality and availability of the power lines themselves. In some cases, power lines are too old or not well-maintained, which leads to poor signal quality and reduced reliability. Additionally, PLC is subject to interference from other electrical devices, which further degrades the quality of the communication signal. On the other hand, the length of the aerial cable, in rural networks, can generally be extended to 1 km when the number of charges is very simple [46].

RF technology is used to provide communication services in rural areas and is particularly useful in areas where power lines do not exist or are not reliable. Furthermore, RF provides high-speed internet access and other communication services to remote and underserved communities, even in areas where traditional infrastructure is lacking. However, wireless technologies are subject to signal degradation and interference from environmental factors, such as weather, terrain, and foliage. These sparsely populated remotely controlled areas are less likely to cause distortions, which explains the low number of paths.

Overall, the choice between PLC and RF technologies will depend on the specific needs and conditions of a given rural environment. A careful consideration of factors such as the existing infrastructure, signal quality, and reliability is the best choice for a rural environment. Both technologies may also be used to provide an uninterrupted and reliable communication network. The typical distance between a smart meter and a data concentrator in a rural environment varies depending on various factors, such as the communication technology used, terrain, vegetation, and the presence of obstacles or interference. For a PLC system, the distance can range from a few hundred meters to several kilometers, depending on the quality of the power lines and the presence of electrical devices or interferences.

### 3.2. Urban Environment

The urban environment refers to an area characterized by a high-density population, a diversity of used devices, and a concentration of human-built infrastructure and services such as transportation systems, housing, commercial buildings, and cultural institutions. It is often characterized by a high concentration of wired and wireless communication infrastructure, providing residents with a wide range of communication options.

First, in an urban environment, by using the existing power grid, PLC is used to provide high-speed connectivity without the need for additional infrastructure or cables and is also used to support specific communication services, such as home automation, smart grid applications, and remote monitoring. However, PLC has some drawbacks compared to other communication technologies. In fact, the quality and reliability of a PLC signal is affected by noise, electrical interference on the power grid, and other factors such as distance, interference from other electrical devices, and the quality of the power grid infrastructure. In addition, PLC is subject to regulatory and safety requirements related to power grid operations [47].

Second, RF communication plays a crucial role in enabling smart grid applications in urban environments. In fact, RF technology enables communication between different components of the grid, such as meters, sensors, and control systems. Therefore, they provide a range of services, such as remote meter reading, demand responses, and real-time monitoring and control of the grid. For example, AMI uses RF communication to enable two-way communication between smart meters and utility companies, allowing real-time monitoring of electricity usage, besides the remote disconnection and reconnection of service. However, it has several limitations that affect its reliability and performance in terms of the SNR and communication range. Some of the key limitations include the interference with other wireless devices, buildings, and other physical obstacles, as well as signal attenuation due to trees and other objects leading to reduced signal power and range.

Therefore, both RF communication and PLC provide redundant or complementary communication services and multiple communication paths for smart meter data, ensuring reliable and secure communication even in the presence of interferences or other disruptions. In urban environments, smart meters and data concentrators are designed to work within a range of a few hundred meters. This range is usually sufficient for most urban environments, where buildings, other structures, and electric network topology can often obstruct the transmission of wireless and wired signals.

### 3.3. Industrial Environment

The industrial environment presents several challenges that affect the effectiveness and the reliability of communication systems. The AMI applications include the use of various sensors, communication devices, and control systems that enable the monitoring, control, and optimization of energy production, transmission, and consumption. Therefore, the main challenge of AMI applications in the industrial environment is the presence of electromagnetic interference (EMI) which is caused by various sources, such as electrical motors and welding equipment. Consequently, reliable and secure communications are designed to provide access control to ensure data confidentiality and integrity.

In AMI applications, on the one hand, PLC technology enables communication between SMs and the DC in order to collect accurate and real-time energy consumption data. However, it faces challenges due to electrical noise, interferences from other devices, and varying signal strengths caused by the different lengths and qualities of power lines [48,49]. On the other hand, RF technology enables communication without the need for physical connections or cables, reducing the cost and complexity of installations. Moreover, IEEE 802.15.4g operates over longer distances, making it suitable for large industrial facilities or remote locations. However, it faces challenges in industrial environments, such as signal interference from other devices, multipath signal propagation, and attenuation due to more obstacles being present, such as walls and metal structures [3,50].

Overall, the specific choice of the communication technology for AMI applications depends on several factors, such as the specific requirements of the industrial environment, the availability of power lines, and the overall cost and performance of the communication system. In this case, the range between the SM and DC can be reduced by the presence of machinery, equipment, or other structures that can obstruct the transmission of wireless signals. In general, in an industrial environment, the distance is likely to be less than in an urban environment, and may require more sophisticated communication technologies to ensure reliable and efficient communication.

### 3.4. Proposed Methodology

**Figure 8:**
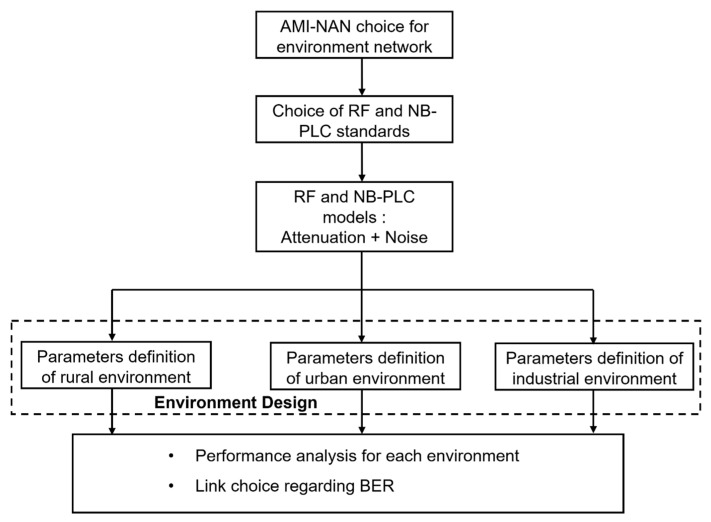
Proposed methodology adopted to study the environmental impact of RF and PLC for AMI.

To ensure a reliable link between the SM and DC in a well-defined area, the choice of the communication technology, either RF or NB-PLC, should be based on several parameters. These parameters can be set to evaluate and compare the performance of each technology. Figure 8 presents the proposed methodology to study the environmental impacts of RF and PLC for AMI. The solution design in this paper focuses on the definition of the characteristics of parameters of RF and NB-PLC channels for distinct geographical areas. In fact, the design concerns the distance between the SM and DC, the number of paths for wireless links, the length of the wired link, and the noise model of each link.

The methodology is summarized in four steps:

- The choice of RF and PLC standards in compliance with the AMI-NAN system: The authors study the choice of Wi-SUN for RF and G3-PLC for NB-PLC. In fact, for the RF link, Wi-SUN allows utilities to monitor and control the electrical grid more efficiently, leading to improved energy management and reliability. Moreover, it supports mesh networking, allowing devices to communicate with each other via multiple paths. For the PLC link, IEEE 1901.2 is a standard for narrowband orthogonal frequency division multiplexing (NB-OFDM) PLC technology. The use of OFDM, which is known for its ability to handle multipath propagation and mitigate the effects of noise and interference, makes communication robust in challenging environments.
- The choice of models for RF and PLC channels: The authors outline the key characteristics of wireless and narrowband power line systems. A bottom-up approach is used for the NB-PLC channel. For the attenuation of the RF channel, the Rayleigh fading model is adopted as it shows the effects of small-scale fading of non-line-of-sight reproduction in wireless communications.
- The environment model design: The authors focus on the environment model design to characterize the RF and NB-PLC channels in distinct geographical environments—rural, urban, and industrial. These characteristics play a crucial role in shaping the design and effectiveness of the AMI system.
- Analysis of performances regarding the BER and link selection for each environment: The authors compare NB-PLC and RF performances for the following three environments, rural, urban, and industrial.

## 4. Simulation Results

### 4.1. OFDM Communication System

**Figure 9:**
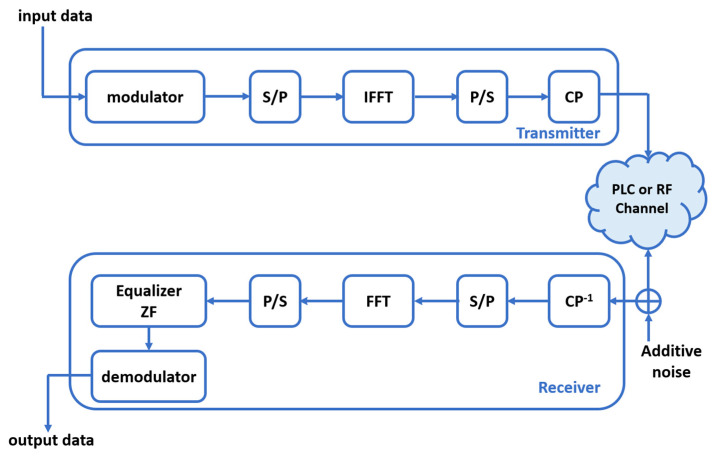
Block diagram of the OFDM system.

This section deals with a simulation model of the OFDM communication system, as described in Figure 9, regarding RF and NB-PLC links. As depicted in Figure 9, the data are processed using the IFFT algorithm, which converts the time-domain signals into frequency-domain signals. This operation splits the signal into multiple subcarriers which are assigned to specific data symbols. According to the selected standards, pilot symbols are added for channel estimation and equalization purposes. Furthermore, when transmitting data between the SM and DC, an appropriate modulation scheme is crucial. The choice of modulation directly impacts the efficiency and reliability of data transmission. At the receiver side, a zero forcing (ZF) equalizer is used in order to mitigate the effects of channel distortion and interference. The performances of simulated OFDM communication chains are evaluated by computing the BER for each value of the signal-to-noise ratio (SNR), reflecting the accuracy of data transmission and representing the ratio of erroneous bits to the total transmitted bits. All simulations are implemented on Matlab tools.

### 4.2. Simulation Parameters

To establish communications between the SM and DC, the authors opt to use the Wi-SUN standard for RF-based communications and the IEEE 1901.2 standard for NB-PLC communications. These standards provide the necessary specifications and guidelines for implementing the communication protocols in AMI systems, ensuring compatibility and reliable data exchange. For NB-PLC, according to the IEEE 1901.2 standard, the OFDM symbol contains a 256-point IFFT adjoined by a cyclic prefix (CP) corresponding to 30 samples. These samples are used to remove the inter-symbol interference (ISI) which is created by the channel effect. For RF, parameters are selected by the authors according to the IEEE 802.15.4g MR-OFDM standard under option 2. They assume that 1 OFDM symbol is the size of 64 subchannels and a CP comprising 16 symbols.

As the OFDM signal is transmitted through the RF or NB-PLC channel, it encounters reflections, delays, and attenuation due to the varying characteristics of the communication environment. To mitigate the effects of multipath fading, channel distortion, and ISI, a zero forcing (ZF) equalizer is implemented in the OFDM system.

As far as the noise is concerned, AWGN, cyclostationary impulsive noise, and Gaussian mixture impulsive noise (GMM) are modelized for wired and wireless communication channels. The specific selection of the noise model for each channel and each area is outlined in Table 3.

The environment design in this paper characterizes RF and NB-PLC channels in distinct geographical areas: rural, urban, and industrial. These characteristics are mandatory to correctly design the AMI system. Therefore, the authors define the key parameters specific to each environment, including (1) the number of paths for wireless links, (2) the length of the wired link, and (3) the noise model associated with each link. Regarding NB-PLC, the authors choose to implement three branches between the SM and DC.

First, in rural environments, the smart metering system is typically designed to cover larger areas and longer distances between devices. This requires the use of higher-power communication technologies, such as NB-PLC or RF, to ensure reliable communication over longer distances. In this paper, the authors use 1 km as a distance between the SM and DC. For the RF communication system, the distance also varies depending on various factors, such as the presence of physical obstacles. This variation is modeled by the RF path number. Therefore, the authors choose the electrical line length that is greater than 200 m and the path number that is equal to 5 with AWGN. Second, in urban environments, the AMI system is typically designed to handle a larger number of connected devices compared to rural environments. This system is designed to operate over short distances and with higher-power communication technologies, NB-PLC or RF, due to the higher density of devices and potential for interferences. In this paper, the authors use 200 m as the distance between the SM and DC. For NB-PLC, and the authors choose the electrical line length between 30 and 50 m with cyclostationary impulsive noise. For RF, the path number is between 10 and 20 with GMM noise and/or AWGN. Third, in industrial environments, the AMI system is typically designed to also handle a large number of devices in complex and noisy environments. To ensure reliable communication, the electrical line length is less than 15 m, and the noise is cylostationary for NB-PLC. The path number of the RF link is greater than 40 and the noise is modeled with GMM as presented in Equation (8). In Table 3, the authors summarize the characteristic parameters of each communication link, RF and NB-PLC, for the three environments described above.

**Figure 10:**
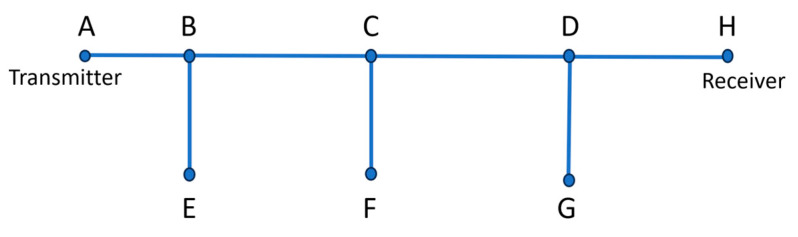
Electrical topology network.

In this paper, the authors choose a topology network with 3 branches between the transmitter A and the receiver H, as shown in Figure 8, where AB, BC, CD, and DH are the lines of the electrical network, whereas E, F, and G are the connected loads whose behaviors are depicted in Figure 10.

### 4.3. Rural Environment

**Figure 11:**
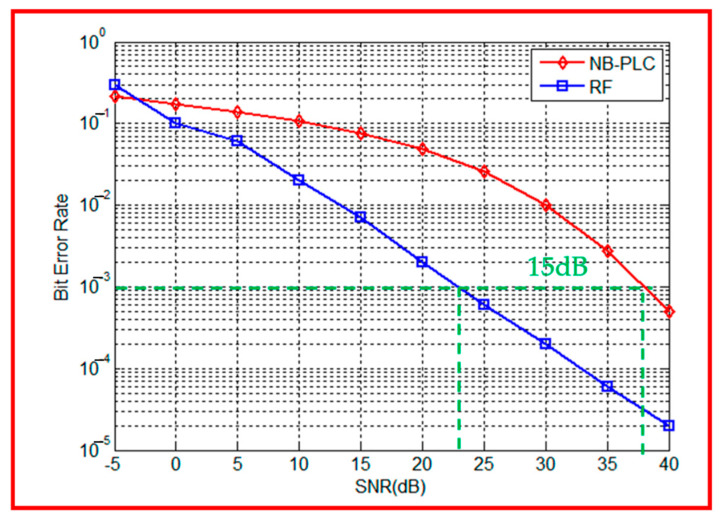
RF vs. NB-PLC performances for a rural environment.

Figure 11 illustrates the performance of the communication chain using RF and NB-PLC links in a rural environment. The simulation parameters for this scenario are summarized in Table 3. The distance between the SM and DC is set to 1 km. For the NB-PLC link, the electric line is divided into three branches, as depicted in Figure 10. Each branch of the NB-PLC line has a length of 200 m. In the simulation setup, the RF path number is equal to 5, indicating the presence of multiple RF paths. The noise model utilized for both RF and NB-PLC links is AWGN.

Figure 11 demonstrates that the RF link outperforms the NB-PLC link in terms of the BER. This is attributed to the rural environment being characterized by a low number of obstructions. With a bit error rate (BER) equal to 10^−3^, the RF link exhibits a higher performance compared to the NB-PLC link, with a difference of approximately 15 dB, which means that to have the same BER = 10^−3^, a higher SNR is required for NB-PLC with respect to RF. Therefore, transmitting data over the RF link is more suitable for rural environments.

In order to provide extensive network coverage for nodes at greater distances, multi-hop operations are likely to be employed in rural areas, where the population is sparse, and the number of obstructions is low. Considering this scenario, the RF technology emerges as a suitable communication solution to simplify information exchange between the SM and DC within this type of environment.

### 4.4. Urban Environment

The authors simulate the performance of both links in an urban environment for two scenarios. In the first scenario, the RF path number is equal to 20 with AWGN, the NB-PLC line length is set to 20 m, and the noise is cyclostationary impulsive noise.

**Figure 12:**
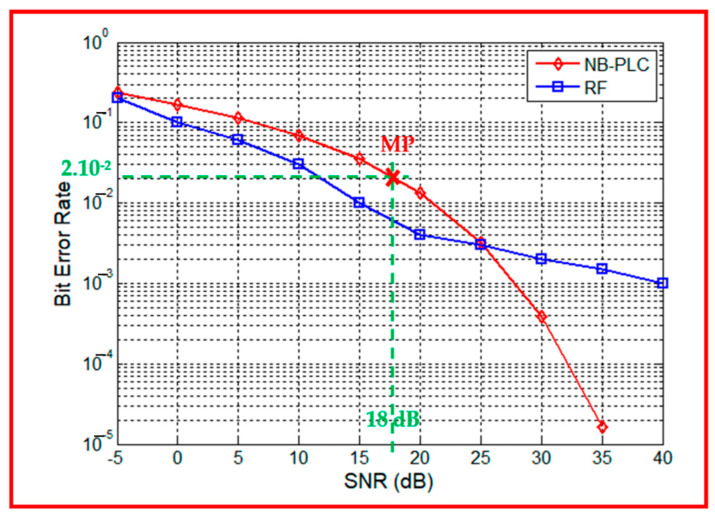
RF vs. NB-PLC performances in an urban environment: 1st scenario for a dense urban environment and a measurement point (MP (X)).

On the one hand, Figure 12 illustrates that, for SNR values lower than 25 dB, the RF link outperforms the NB-PLC link. There is an approximate 7 dB gap observed for a BER of 10^−2^. This indicates that despite the environmental density, the wireless link performs better than the wired link. Thus, the electrical network topologies within buildings significantly attenuate the signal transmitted between the SM and DC.

On the other hand, for an SNR greater than 25 dB, the NB-PLC link outperforms that of the RF link, with an approximate 13 dB gap observed for a BER of 10^−3^. These results demonstrate that despite the low level of noise, the BER of the wireless link does not decrease considerably.

From [51], the authors select experimental sites in Korea according to the customer type, and measure signal and noise powers at different measurement points. Communication performances are observed and analyzed for practical AMI field environments.

In fact, a series of experimental measurements were conducted in a commercial environment [51]. The results are presented in terms of PER, which is expressed as a function of the BER in Equation (9).

(9) $$PER=1-{\left(1-BER\right)}^{packet\_length}\;$$

The authors selected a measurement point (MP) characterized by a distance of 75 m between the SM and DC, corresponding to a high-density urban environment in the case of this paper. At this MP, the PER is equal to 90% and the SNR is equal to 18 dB. By applying Equation (9), and with a packet_length = 100 bytes, the obtained BER is 2.10^−2^, which corresponds to an SNR = 18 dB. Figure 12 presents this measurement point and confirms the simulation results obtained in this case.

To investigate the impact of the path number for the RF channel and the line length for the PLC channel on the performance of the communication system, a second scenario is defined. It represents an urban area characterized by moderate congestion and noise. In this scenario, the authors decrease the path number parameter from 20 to 10 and increase the line length from 20 m to 50 m.

**Figure 13:**
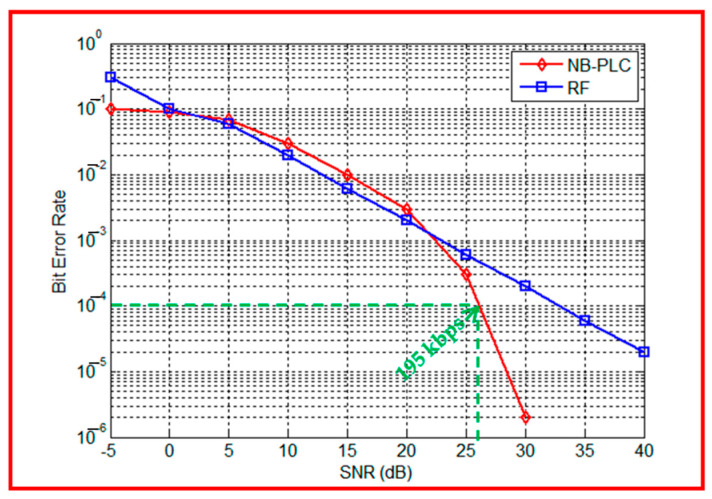
RF vs. NB-PLC performances in an urban environment: 2nd scenario for a moderately dense urban environment, with an example point that gives a data rate equal to 195 kbps.

Figure 13 shows that for SNR values less than 25 dB, the BER performances are similar for both channels. However, for SNR values greater than 25 dB, the PLC is more suitable. Thus, buildings in urban areas attenuate the RF signal, despite the complexity of the electrical network.

Furthermore, the authors compute the NB-PLC data rate at a BER equal to 10^−4^. According to Figure 13, it is equal to 195 kbps, which corresponds to the average data rate value, which is 200 kbps in the G3-PLC standard, depicted in Table 1.

Compared to the rural area, it can be observed that an increase in the number of paths and the complexity of the network topology impact the transmission performance of the channels in urban areas.

### 4.5. Industrial Environment

**Figure 14:**
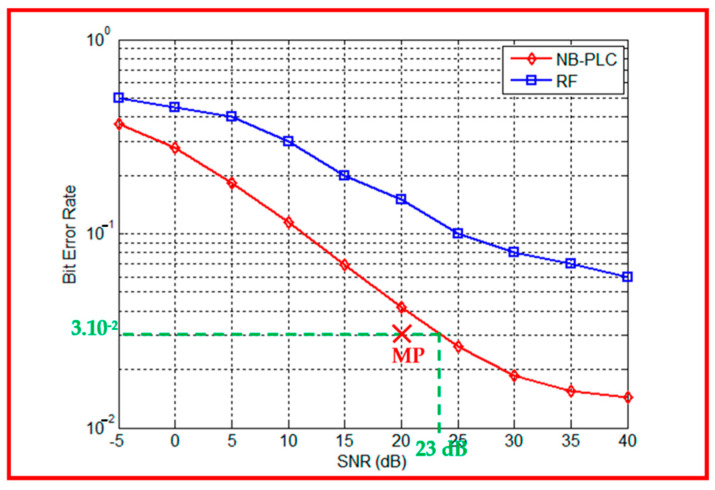
RF versus NB-PLC performances in an industrial environment, and a measurement point (MP (X)).

In order to supervise physical equipment, the industrial network represents the most suitable system since it works on studying different interlinked equipment. Figure 14 shows the performance of RF and NB-PLC in terms of the BER. For the RF link, the path number is equal to 40 and the noise is GMM and uses Equation (9), where π_1_ = 0.99, π_2_ = 0.01, $\sigma_{1}^{2}$ = 0.001, and $\sigma_{2}^{2}$ = 100. For the NB-PLC link, the length line is equal to 10 m and the noise is cyclostationary impulsive. Dually to the rural case, for the industrial environment to have the same BER value, a lower SNR value is required with NB-PLC if compared to RF.

The authors demonstrate that despite the high connection density, the presence of light loads, non-linear loads, and various distribution types in the industrial area, the NB-PLC is more performant than the RF link. In fact, NB-PLC systems are designed to handle and mitigate such noise, ensuring reliable and robust communication even in the presence of these disturbances. Furthermore, NB-PLC signals can penetrate through various obstacles and materials commonly found in industrial areas, such as walls, metal structures, and machinery. This allows for better signal propagation and coverage compared to RF.

The authors selected another measurement point (MP) from [51]. Its parameters are in accordance with the conditions of the modelized industrial environment in this paper as shown in Figure 14. At this MP, the PER is equal to 95% and the SNR is equal to 20 dB. Applying (9) returns a BER equal to 3.10^−2^, which corresponds to an SNR equal to 23 dB, according to Figure 14. A difference of 3 dB is noticed between simulation results and experimental measurements confirming the reliability of the industrial environment model and the simulation results.

### 4.6. Discussion

The proposed method allows us to understand what would be the expected from a BER and thus the preferred medium based on the consideration on the environment and the value of an SNR. Based on the simulation results, the following considerations can be summarized:

- Rural Environment: Simulation results suggest that the RF link demonstrates better performance in rural areas. Since they typically have lower population density and less electromagnetic interference, the use of the RF link can be more feasible and provide reliable communication with better performance. Interestingly, PLC can be a valid option even in low-density rural areas if repeaters can be installed. In this viewpoint, the protocol choice is crucial. The G3 or PRIME protocol has a mechanism to promote simple nodes to relay nodes and automatically build networks. This kind of protocol can be exploited in order to obtain a strong interconnected and reliable PLC network.
- Industrial Environment: Simulation results indicate that NB-PLC can be more suitable for industrial areas, likely due to the advantages mentioned earlier, such as the presence of abundant power lines, signal penetration, interference immunity, and noise rejection.
- Urban Environment: The choice between the RF link and NB-PLC for communication between the SM and DC in an AMI system is more complicated and it depends on the density of the area. The decision between them in this scenario depends on other factors, such as infrastructure availability, cost considerations, and specific application requirements. The communication between the SM and DC is affected by multipath propagation and external interferences. This limits network performances in terms of reliability and availability. Therefore, additional schemes, such as repeaters or error correcting codes, are needed to improve the link reliability requirements.

## 5. Conclusions

This paper analyzes the impacts of different environment types, including rural, urban, and industrial environments, on NB-PLC and RF communication technologies applied between the SM and DC in an AMI system. The authors propose optimal communication solutions and highlight the advantages of using specific technologies in each environment. The obtained results show that in rural environments, the RF link is more suitable for transmitting data than PLC. The lower population density and reduced electromagnetic interference in rural areas make wireless communication a feasible and reliable option. However, in dense urban areas with obstructions, this study recommends NB-PLC when the noise level is low. NB-PLC utilizes existing power lines, providing a reliable communication link in urban environments with fewer obstacles. Finally, for industrial areas, the authors identify NB-PLC as the preferred choice for communication between the DC and SM. The inherent advantages of NB-PLC, such as signal penetration through obstacles, noise immunity, and the availability of power lines, make it more performant than the RF links in industrial settings.

The aim of this paper is to propose a model that can be adapted to the real world based on the actual structure of a network. This model aims to understand the extent of attenuation and make informed decisions regarding the implementation of infrastructure.

In order to enhance system reliability and overcome limitations, the authors suggest implementing a hybrid system that combines both NB-PLC and RF technologies. By utilizing the best aspects of each technology, a hybrid approach can optimize the communication link and ensure the lowest attenuated transmitted signal, resulting in improved overall performance.

## Figures and Tables

**Table 1:** Wired and wireless technologies for LV smart metering applications.

Technology	Frequency Band	Average Data Rate	Average Latency	Standards
NB-PLC	3–500 kHz	200 kbps	150 ms	PRIME, G3-PLC/IEEE 1901.2, ITU-T G.hnem
BB-PLC	2–30 MHz	100 Mbps	200–400 ms	IEEE1901, ITU-T G.hnem
Optical Fiber	>1 THz	Up to 10 Gbps	5 μs	Ethernet 10GBASE-SR
WiMAX	2–11 GHz	75 Mbps	10–50 ms	IEEE 802.16, IEEE 802.16d
RF Mesh	900 MHz	10–100 kbps	700 ms	IEEE 802.11, IEEE 802.15
LTE	900 MHz	384 kbps	100 ms	LTE-M
3G	800 MHz	Up to 14.7 Mbps	120 ms	UMTS

**Table 2:** Time percentage of noise regions.

Region	Percentage
R1	70%
R2	20%
R3	10%

**Table 3:** Parameters of characteristics.

Area	SM-DC Distance	RF Path Number	RF Noise Model	NB-PLC Line Length	NB-PLC Noise Model
Rural	1 km	5	AWGN	>200 m	AWGN
Urban	80 m–200 m	10–20	AWGN/GMM	20–50 m	Cyclostationary Impulsive Noise
Industrial	50 m	40–50	GMM	<15 m	Cyclostationary Impulsive Noise

## Data Availability

Data are contained within the article.
